# Enteropathogens Associated with Acute Diarrhea in Children from Households with High Socioeconomic Level in Uruguay

**DOI:** 10.1155/2015/592953

**Published:** 2015-03-12

**Authors:** Gustavo Varela, Lara Batthyány, María Noel Bianco, Walter Pérez, Lorena Pardo, Gabriela Algorta, Luciana Robino, Ramón Suárez, Armando Navarro, María Catalina Pírez, Felipe Schelotto

**Affiliations:** ^1^Departamento de Bacteriología y Virología, Facultad de Medicina, Universidad de la República, Alfredo Navarro 3051, 11600 Montevideo, Uruguay; ^2^Departamento de Pediatría, British Hospital, Avenida Italia 2420, 11600 Montevideo, Uruguay; ^3^Departamento de Laboratorio Clínico, British Hospital, Avenida Italia 2420, 11600 Montevideo, Uruguay; ^4^Departamento de Salud Pública, Facultad de Medicina, Universidad Nacional Autónoma de México, Ciudad Universitaria, Avenida Universidad 3000, 04510 Ciudad de México, DF, Mexico; ^5^Departamento de Pediatría, Facultad de Medicina, Universidad de la República, Centro Hospitalario “Pereira Rossell”, Boulevard Artigas 1550, 11600 Montevideo, Uruguay

## Abstract

Infectious diarrhea, a common disease of children, deserves permanent monitoring in all social groups. To know the etiology and clinical manifestations of acute diarrhea in children up to 5 years of age from high socioeconomic level households, we conducted a descriptive, microbiological, and clinical study. Stools from 59 children with acute community-acquired diarrhea were examined, and their parents were interviewed concerning symptoms and signs. Rotavirus, adenovirus, and norovirus were detected by commercially available qualitative immunochromatographic lateral flow rapid tests. *Salmonella, Campylobacter, Yersinia*, and *Shigella* were investigated by standard bacteriological methods and diarrheagenic *E. coli* by PCR assays. We identified a potential enteric pathogen in 30 children. The most frequent causes of diarrhea were enteropathogenic *E. coli* (EPEC), viruses, *Campylobacter, Salmonella*, and Shiga-toxin-producing *E. coli* (STEC). Only 2 patients showed mixed infections. Our data suggest that children with viral or *Campylobacter* diarrhea were taken to the hospital earlier than those infected with EPEC. One child infected with STEC O26 developed “complete” HUS. The microbiological results highlight the importance of zoonotic bacteria such as atypical EPEC, *Campylobacter*, STEC, and *Salmonella* as pathogens associated with acute diarrhea in these children. The findings also reinforce our previous communications about the regional importance of non-O157 STEC strains in severe infant food-borne diseases.

## 1. Introduction

Infectious diarrhea continues to be a health burden worldwide, especially in children living in developing countries. It is estimated that in these regions it is responsible for 2.5 million infant deaths annually, with a mortality rate of 4.9 per 1,000 children and an annual incidence of 3 episodes per child among children under 5 years of age [1, 2].

In developed countries the mortality from diarrhea is relatively low but morbidity is not so different from that seen in developing countries. Due to the relatively high incidence, the social impact and health costs are similar in both situations [3, 4].

*Salmonella; Shigella; Yersinia enterocolitica; Campylobacter; Vibrio cholerae*; diarrheagenic* Escherichia coli* pathotypes (DEPs), and viruses represent leading causes of infantile acute diarrhea in both developing and developed countries. DEPs comprise several categories: enteropathogenic* E. coli* (EPEC), that can be further classified into typical EPEC (tEPEC) and atypical EPEC (aEPEC) depending on the presence or absence of the EAF plasmid (*E. coli* adherence factor); Shiga-toxin-producing* E. coli* (STEC); enterotoxigenic* E. coli* (ETEC); enteroaggregative* E. coli* (EAEC); and enteroinvasive* E. coli* (EIEC). However, its relative importance is different in high- and low-income populations [5–10].

DEPs,* Campylobacter*, and most viruses are not routinely investigated as enteric pathogens in most clinical laboratories worldwide; thus, their importance in infant community-acquired diarrhea is generally underestimated [2].

In our country, most public or private microbiology laboratories only perform the detection of* Salmonella*,* Shigella*, and some viruses in feces from children with diarrhea [11, 12].

Our research group has conducted detailed studies about the etiology of diarrheal diseases. However, those studies have been mainly performed in children from households with low or very low socioeconomic level [4, 13, 14].

This study aimed to evaluate the association of DEPs,* Salmonella*,* Shigella*,* Yersinia enterocolitica*,* Campylobacter*, and viruses with cases of acute diarrhea in a subset the children living in households with high socioeconomic level. The clinical characteristics of the illness related to the most common agents were also analyzed.

## 2. Methodology

### 2.1. Study Design

Between January 2010 and March 2011 we conducted a descriptive, microbiological and clinical study involving children ≤5 years of age, with acute community-acquired diarrhea living in households with high socioeconomic level.

Diarrhea was defined as the passing of three or more liquid or loose stools (which take the shape of the container) within a 24-hour period.

Children with persistent diarrhea (more than 15 days of duration); those who were receiving antibiotics or had been hospitalized in the 30-days period before the episode of acute diarrhea; or children enduring gastrointestinal disorders as celiac disease, allergy to cow's milk, or inflammatory bowel disease were not included.

### 2.2. Setting

We studied children ≤ 5 years of age seeking medical care in clinics or emergency room of a private health institution, British Hospital, a 120-bed tertiary hospital with medical and surgical pediatric specialties. Children receiving medical care at the British Hospital often come from high-income households.

We included only those children in whom the stool sample was available during the visit. The study was approved by the technical commission of the British Hospital and by the School of Medicine Ethical Committee (Universidad de la República; file number 071140-000323). Verbal agreement to participate in the study was initially received from patient's parents through their physician.

### 2.3. Stool Investigations

Only one sample per child was analyzed. We performed macroscopic observation to trace the presence of blood, mucus, pus, or other abnormal element and searched for fecal leukocytes (FL) in smears stained with methylene-blue and then microscopically examined under high-dry power (×400).

### 2.4. Viruses

The assays were performed at the laboratory of the British Hospital by using commercially available qualitative immunochromatographic lateral flow rapid tests for norovirus, rotavirus, and adenovirus (RIDA Quick Norovirus and RIDA Quick Rotavirus/Adenovirus Combi-Biopharm AG, Damstadt, Germany), respectively. The instructions from the manufacturer were followed.

### 2.5. Bacteria

Stool samples were placed in Cary-Blair medium (Difco, Becton, Dickinson) and chilled and transported to the laboratory of the Bacteriology and Virology Department, for detecting* Salmonella*,* Shigella*,* Campylobacter*,* Yersinia enterocolitica*, and DEPs according to previously described procedures [4, 14–16].

Briefly, selective and differential culture media (*Salmonella-Shigella* agar, MacConkey agar, Sorbitol MacConkey agar, and* Yersinia* selective agar) (Difco, Becton, Dickinson) were used for isolating* Salmonella, Shigella*, DEPs, and* Yersinia* species. Tetrathionate broth (Difco, Becton, Dickinson) incubated at 42°C, peptone-sorbitol bile broth (PSB) incubated at 4°C, and cefixime-tellurite/tryptic soy broth (CT-TSB) (Oxoid, Basingstoke, UK and Difco, Becton, Dickinson, resp.) incubated at 37°C were used as enrichment media for* Salmonella, Yersinia*, and STEC, respectively. For* Campylobacter* detection, feces were cultured at 42°C in a microaerophilic environment on selective medium prepared with Brucella agar base (Difco, Becton, Dickinson), hemin, sodium metabisulfite-ferrous sulfate-sodium pyruvate (Sigma-Aldrich), sheep blood, and the Campylosel mixture (bioMéreieux, Marcy l'Etoile, France): cefoperazone, colistin, vancomycin, and amphotericin B [17].

After incubation for 24 h at 37°C, up to 40 colonies with typical* E. coli* morphology were subjected to PCR using the primers shown in the Table 1 to detect the following genes:* stx*1 and* stx*2 of STEC;* eae* of enteropathogenic* E. coli* (EPEC) and some STEC strains;* ipaH *of enteroinvasive* E. coli* (EIEC) and* Shigella*;* elt* and* estA* of enterotoxigenic* E. coli* (ETEC); and pCVD432 of EAEC [4, 15].

Bacterial colonies were pooled for DNA extraction in 150 *μ*L of sterile water, boiled for 10 min, and then centrifuged at 13,000 rpm for 10 min. A 2,5 *μ*L aliquot of this supernatant was added to 22,5 *μ*L of the PCR mixture reaction (50 mM KCl, 10 mM Tris-HCl [pH 8.3], 1.5 mM MgCl_2_, and 2 mM of each deoxynucleoside triphosphate), 1.25 U of* Taq* DNA polymerase (HybriPol Bioline, UK), and each primer pair (SBS Genetech Co., Ltd) shown in Table 1. The reactions were run in a thermal cycler (Gene Amp PCR System 2700 Applied Biosystem) with the following cycling conditions: 94°C for 5 min; 35 cycles of denaturation at 95°C for 1 min; and annealing (see Table 1 for annealing temperature) for 1 min and extension at 72°C for 1 min, followed by a final extension at 72°C for 10 min. PCR products were visualized after electrophoresis in 2% agarose gels in 0.5X TBE buffer and ethidium bromide staining.

STEC isolates were further characterized by the presence of the* ehxA* and* eae* genes, according to previously described PCR reactions [16]. Variants of* eae* genes present both in EPEC and STEC recovered strains were also determined by PCR assays [4, 17, 18].

In EPEC isolates (*eae* positive and* stx*1/2 negative), the presence of the* bfp* gene was detected by PCR as we described previously, using the primer pairs shown in the Table 1 [16].

Variants of* bfp* gene were also determined by PCR, as we described previously [4].

The DEPs strains detected were confirmed as being* E. coli* by using the API 20 E system (Bioméreieux, Marcy l'Etoile, France) and conventional biochemical assays.

Colonies suspected to be of* Salmonella*,* Shigella*,* Campylobacter*, or* Yersinia* genera were studied according to classical procedures described previously [13–15].

DEPs serotyping was provided by Dr. Armando Navarro from the Universidad Autónoma de México [18].

*Salmonella* serotyping was done at the Centro Nacional de* Salmonella* (CNS, Department of Bacteriology and Virology, Institute of Hygiene).

Antimicrobial susceptibility to amikacin, ampicillin, ceftriaxone, cefuroxime, ciprofloxacin, cloramphenicol, colistin, gentamicin, nalidixic acid, nitrofurantoin, streptomycin, tetracycline, and trimethoprim-sulfamethoxazole (Oxoid Ltd., Basingstoke, Hampshire, UK) was established according to Clinical Laboratory Standards Institute (CLSI) guidelines [19].

### 2.6. Interviews

The children were seen by a pediatrician. Their parents were enquired about signs, symptoms, and other relevant clinical or epidemiological information by using a standard questionnaire when the child was enrolled in the study. The data collected were age, gender, macroscopic characteristics of stools, presence of fever and/or vomiting, number of stools, and duration of diarrhea in hours from the beginning to the time of the visit.

The socioeconomic level was assessed using the modified Graffar scale. Information on housing, educational level, and occupation of the parents and income was collected. Aggregate scores were classified into five social class categories ranging from very high I (4–6 points) to very low V (17–20 points). We considered the children from households within categories I or II as having high socioeconomic conditions [20].

Height and weight were determined according to standard anthropometric methods. In order to analyze the nutritional status, the weight and height of each child were compared with those of same aged boys and girls measured in the National Health and Nutritional Examination Survey in the USA (NHANES). The degree of dehydration was estimated clinically by analyzing general appearance, eyes, mucous membranes, and tears.

The clinical outcome was monitored by telephone interviews.

## 3. Results

Ninety-eight children ≤5 years of age were taken to British Hospital for diarrhea during the study period. However, only 59 (60%) met all inclusion criteria. Most excluded children that had acute diarrhea (85%) were not considered because stool samples were not available during the medical visit. The remaining 15% presented associated or underlying intestinal disease (e.g., allergy to cow's milk, lactose intolerance, and malformations).

### 3.1. Demographic and Socioeconomic Characteristics

Thirty-three (58%) of 59 were male children. The average age was 11 months with a range from 1 month to 5 years. All the analyzed children were from households belonging to categories I or II of the modified Graffar scale.

### 3.2. Microbiological Aspects

**Figure 1:**
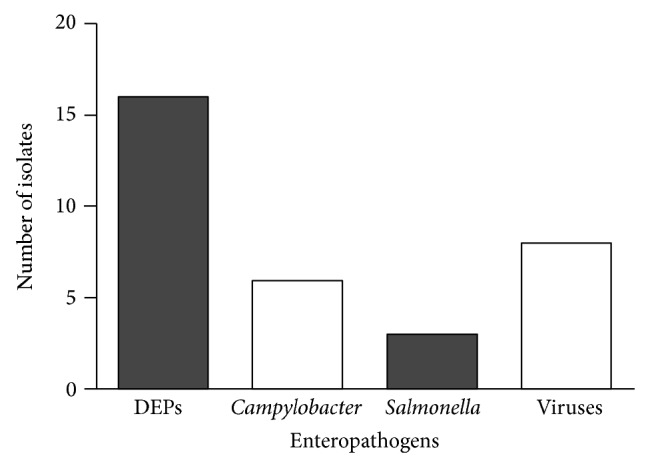
Enteropathogens detected in children with acute diarrhea from households with high socioeconomic level in Montevideo, Uruguay.

In 30 of 59 enrolled children (51%) we detected potential enteric pathogens. The 32 agents identified were the following: rotavirus 3; adenovirus 3; norovirus 2; DEPs, 16 isolates; 5 isolates of* Campylobacter*; and 3 isolates of* Salmonella*. Viruses were thus found in 8 (13%) cases and bacteria in 24 (40%) (see Figure 1).

*Campylobacter* was recovered as single pathogen in 5 children, while viruses were detected as single agents in 6 of them.

Four out of the five* Campylobacter* isolates were identified as* C. jejuni* and the remaining corresponded to* C. coli.*

Two serovars of* Salmonella* were recovered:* Salmonella enterica* subsp.* enterica* serovar Enteritidis, 2 isolates; and* Salmonella enterica* subsp.* enterica* serovar Typhimurium 1.

The most frequently isolated bacterial pathogen was diarrheagenic* E. coli* followed by* Campylobacter*.

Table 2 shows the characteristics of DEPs recovered strains.

The distribution of* eae* subtypes in aEPEC isolates was as follows: O26, *β*; O28ac, *γ*1; O34, *θ*; O103, *β*1; O125, nontypable; and ONT, *κ*.

The 3 tEPEC recovered strains showed* bfp* gene type *β*. Serogroup O86 isolates (2 strains) showed* eae* gene subtype *ι*, while the O26 strain carried subtype *β*.

All STEC isolates were* eae* and* ehx*A positive. Both O26 strains carried* eae* gene subtype *β*1, but we were unable to establish the* eae* subtype of STEC strain O153.

One O26 STEC strain carried* stx*1 gene, and the other one carried both* stx*1 and* stx*2 genes. The O153 STEC strain was only positive for* stx*2.

The 4 EAEC isolated strains were lysine-decarboxylase positive.

Two children showed mixed infections; one was infected with both aEPEC and rotavirus and the other one with adenovirus and EAEC.

We could not recover ETEC, EIEC,* Shigella*, or* Yersinia enterocolitica* isolates.

One aEPEC serogroup O103 strain showed resistance to nalidixic acid; one tEPEC (serogroup O86) was resistant to ampicillin; and one EAEC (ONT) isolate was resistant to ampicillin, tetracycline, and trimethoprim-sulfamethoxazole. The remaining recovered DEPs were susceptible to the all tested antibiotics and so were* Salmonella* isolates.

### 3.3. Clinical Aspects

Selected clinical data of the children with diarrhea due to EPEC (both typical and atypical),* Campylobacter*, and viruses are given in Table 3. Only the clinical findings of children in which a single enteropathogen was detected are shown.

All children were well nourished at the moment of the episode of acute diarrhea.

Overall, the viral infections were characterized by vomiting. In 9 of the 59 studied children we detected fecal leukocytes. In 6 of these children we identified a potential enteropathogen (see Table 3).

Bloody diarrhea and fecal leukocytes were characteristic of* Campylobacter* and* Salmonella* infections.

One child infected with STEC (serogroup O26; genotype:* sxt*1/2,* eae* subtype *β*1, and* ehxA* positive) had bloody diarrhea and after 20 days developed “complete” HUS with microangiopathic hemolytic anemia, thrombocytopenia, and acute renal involvement, requiring dialysis in the acute stage. None of the 59 children showed severe dehydration at the time of pediatric consultation, and none required hospitalization for intravenous fluid reposition.

None of the children infected with aEPEC strains evolved to persistent diarrhea.

None of the children infected by* Campylobacter* developed Guillain-Barré syndrome. There were no deaths in this group of children.

## 4. Discussion

Infectious gastroenteritis is one of the most common diseases in humans, with particularly high morbidity and mortality in children younger than 5 years of age. The pathogens involved vary according to mainly the socioeconomic conditions of the analyzed population. Knowledge of the prevalent agents is important to design specific control measures, vaccination strategies, and treatment regimens [1, 2].

In our country and in the region there are no published studies about the etiology of acute community diarrhea in children from households with high socioeconomic level.

In this first local study, investigating 12 agents (EPEC, ETEC, STEC, EAEC, EIEC,* Salmonella*,* Shigella*,* Y. enterocolitica*,* Campylobacter*, rotavirus, adenovirus, and norovirus) allowed to recover a potential enteric pathogen in 52% of the children, a similar figure to that reported previously by Olesen et al. in Denmark [10]. 60% of children ≤ 5 years of age brought to British Hospital for diarrhea during the mentioned period were studied. This private health institution attends 4.000 children and is quite representative of the focused on social group.

Viruses were detected in 13% of these children (see Figure 1). Several studies show that viruses are the leading cause of infantile acute diarrhea, both in developed and developing countries [21, 22]. The role of viruses could be underestimated here, since agents such as astrovirus and sapovirus, well-known diarrheagenic pathogens, were not investigated in this study [23, 24].

With regard to bacterial causes, our findings are in agreement with results of previous studies involving children of high socioeconomic level. Our data suggest that* Campylobacter jejuni* and* Salmonella* are common bacterial gastrointestinal pathogens in this group of children [25, 26].

Neither* Shigella* nor* Yersinia* seems to play an important role as diarrhea agents in these patients. However, a previous study conducted by us that included 95 children ≤ 5 years of age from low socioeconomic-level households suffering from acute diarrhea showed the involvement of these agents in that situation. Using an identical methodology, we recovered one or more potential enteric pathogens in 74% of these children.

The identified agents were rotavirus in 42 cases; DEPs, 40 isolates;* Shigella,* 18 strains; seven isolates of* Campylobacter jejuni*; and 3* Yersinia enterocolitica* strains. Within the set of DEPs, the most frequently found pathotype was EPEC (26 isolates: 15 corresponded to typical and 11 to atypical ones) followed by ETEC (8 strains) and EAEC (6 strains). No strains of STEC or EIEC were recovered. 20% of these children showed mixed infections with 2 or more enteric pathogens [4].

Similar results were obtained in other studies conducted by our group [13, 14].

Several points of interest related to DEPs can be highlighted in this study.

Atypical EPEC was the most commonly isolated category of diarrheagenic* E. coli* in the present study. On the other hand, tEPEC strains were recovered less frequently in this infantile subpopulation (see Table 2).

This finding is consistent with previous observations indicating that infections with aEPEC exceed those with tEPEC in both developing and developed countries [27, 28]. Our results suggest that this trend is also present in Uruguay, especially in children from high-income households.

The reasons for these changes are not well known but could be related to the increased exposure of these children to the animal reservoir of these agents, to changes in eating habits, or in the way of cooking certain foods, among other causes [29].

STEC strains recovered carried genes associated with severe diseases (*stx*,* eae*, and* ehx*A); and one child infected with O26 STEC developed HUS. This is interesting because we have previously isolated STEC strains belonging to non-O157 serogroups from children with HUS from low-income households and also from bovine feces [30].

These findings reinforce our suggestion posed in previous communications about the local participation of non-O157 STEC strains in severe infant diseases and also addresses the importance of performing active surveillance of all forms of HUS [16, 30]. The third STEC recovered strain which belonged to a serogroup not previously isolated in our country or in the region. This fact highlights the value of STEC screening based on the detection of* stx* genes.

Intimin (encoded by* eae* gene) is involved in the binding of EPEC and STEC to enterocytes and provides information about the association of EPEC and STEC with severe diseases. We found a variety of* eae* subtypes, both in EPEC and STEC isolates. This superficial structure could become a target for local and regional vaccine development, so it is important to know the genetic variants present in the circulating strains [31].

The EAEC strains were found second in frequency after aEPEC. EAEC has been identified as a common cause of acute diarrheal illness in children and adults of inpatient and emergency units throughout the United States [32].

EAEC as a pathogen is not preferentially included in clinical diagnostic tests, and the molecular mechanisms underlying pathogenesis are poorly understood. In the absence of a defined virulence marker and considering that all EAEC recovered strains were lysine-decarboxylase positive, its role as enteropathogen for this group of children is debatable [26].

Despite the high number of colonies with typical* E. coli* morphology studied per child, we could not recover ETEC nor EIEC strains. ETEC and EIEC are well-known pathogens, but they are common in children from households with low socioeconomic level. We recovered ETEC and EIEC isolates in previous studies that included children with acute diarrhea from low-income families admitted to the public pediatric hospital “Hospital Pediátrico-Centro Hospitalario Pereira Rossell” in Montevideo [13, 14].

As expected, only 2 (7%) out of 30 children showed mixed infections in the present study. This figure is lower than that we found previously in children with diarrhea from low-income households. In that subpopulation of children the figure was almost 40% [13].

In this study we observed a low level of resistance to antibiotics among DEPs and* Salmonella* strains. Only 3 of 19 tested isolates showed resistance to one or more antibiotics. This can be due to the proper application of the guidelines currently recommended for empirical treatment of children with diarrhea [33]; it may also result from a lower use of antibiotics as a feed supplement for slaughter animals, especially cattle and chickens.

With regard to the clinical findings, our data suggest that children suffering from viral or* Campylobacter* diarrhea were brought to the hospital (emergency room or clinics) earlier than those infected with EPEC. Most of them were taken within 48 hours of disease onset. This may have occurred because most children with viral infection presented profuse vomits from illness onset and most children infected with* Campylobacter* had blood and/or mucus in the stools. Vomits and blood or mucus in the stools could act as alarm signs for the caregivers of these patients, leading them to quickly seek medical attention [4, 26].

Most children infected with viruses showed watery diarrhea. In EPEC infections watery diarrhea was also frequent, although some of these children had bloody diarrhea (see Table 3).

The mechanism by which EPEC can produce bloody diarrhea is not well known. However, the existence of inflammatory elements in the stools of children infected by EPEC has been demonstrated by using sensitive methods for detecting leukocyte lactoferrin [34].

Almost 90% of children infected by* Campylobacter* showed polymorphonuclear leukocytes in feces, while only 20% of those infected by EPEC showed a positive result.

## 5. Conclusions

This study increases our knowledge about the etiology of acute infantile diarrhea.

Viruses,* Campylobacter*, and* Salmonella* were found as common pathogens in this subpopulation of high-socioeconomic level children suffering acute diarrhea. The role of diarrheagenic* E. coli* was also confirmed for EPEC (aEPEC and tEPEC) and non-O157 STEC strains. O26 STEC was found responsible for a case of HUS, a severe childhood disease. Obtained data suggest that children with viral or* Campylobacter* diarrhea were taken to the hospital earlier than those infected with EPEC.

Our results highlight the importance of zoonotic bacteria as pathogens associated with acute diarrhea in this subpopulation of high-socioeconomic level children.

The examination of stool cultures from children ≤ 5 years of age suffering from acute diarrhea and living in households with high socioeconomic level should include tests for viruses,* Campylobacter*,* Salmonella*, EPEC, and STEC. Ideally, identification of EPEC and STEC should be based on detection of the* eae* and* stx* genes, respectively.

## Figures and Tables

**Table 1:** Primers used for amplifying genes of DEPs.

Gene	Primer	Oligonucleotide sequence (5′-3′)	Product length (bp)	Annealing temperature	Reference
*stx1*	VT1-A	CGCTGAATGTCATTCGCTCTGC	302	55°C	[4]
	VT1-B	CGTGGTATAGCTACTGTCACC			

*stx2*	VT2-A	CTTCGGTATCCTATTCCCGG	516	55°C	[4]
	VT2-B	CTGCTGTGACAGTGACAAAACG			

*eae *	EAE-1	GGAACGGCAGAGGTTAATCTGCA	775	55°C	[4]
	EAE-1	GGCGCTCATCATAGTCTTTC			

*bfp *	EP1	AATGGTGCTTGCGCTTGCTGC	326	60°C	[4]
	EP2	GCCGCTTTATCCAACCTGGTA			

*ipa*H	EI1	GCTGGAAAAACTCAGTGCCT	424	55°C	[4]
	EI2	CCAGTCCGTAAATTCATTCT			

pCDV432	432/start	CTGGCGAAAGACTGTATCAT	630	60°C	[4]
	432/stop	CAATGTATAGAAATCCGCTGTT			

*eltA *	LT-A-1	GGCGACAGATTATACCGTGC	696	55°C	[4]
	LT-A-2	CCGAATTCTGTTATATATGTC			

*estA *	STA-1	ATTTTTATTTCTGTATTGTCTTT	176	48°C	[4]
	STA-2	GGATTACAACACAGTTCACAGCAG			

**Table 2:** DEPs strains isolated from children with acute diarrhea from households with high socioeconomic level.

Pathotype	Number of isolates	Serogroup (number of isolates)
aEPEC	6	O26 (1), O28ac (1), O34 (1), O103 (1), O125 (1), and ONT^a^ (1)
tEPEC	3	O86 (2) and O26 (1)
EAEC	4	O3 (2) and ONT^a^ (2)
STEC	3	O26 (2) and O153 (1)

^a^NT, non typable.

**Table 3:** Clinical data of the children with diarrhea by EPEC, *Campylobacter*, viruses or *Salmonella*.

	Pathogens^¶^			
	EPEC^§^ *n* = 8	*Campylobacter* *n* = 5	Virus^*^ *n* = 6	*Salmonella* *n* = 3
Duration^a^				
Between 12 and 48 h	3	4	5	3
Between 72 and 96 h	4	1	1	0
>120 h	0	0	0	0
No data	1	0	0	0
Stool appearance^b^				
Watery	5	1	5	0
Bloody	2	4	0	3
Mucus	0	0	1	3
No data	1	0	0	0
Fever	4	3	3	2
Vomiting	2	1	5	1
FL^c^	2	4	0	3

^¶^Only the clinical findings of children with diarrhea in which a single enteropathogen was detected are shown; ^§^includes both aEPEC and tEPEC isolates; ^*^includes rotavirus, adenovirus, and norovirus.

^a^Number of hours with diarrhea before the visit; ^b^data were provided by the parents/or pediatrician; ^c^fecal leucocytes, defined as PMN cells identified in the stools by methylene-blue stain.
